# Exploring the Potential of Nitric Oxide and Hydrogen Sulfide (NOSH)-Releasing Synthetic Compounds as Novel Priming Agents against Drought Stress in *Medicago sativa* Plants

**DOI:** 10.3390/biom10010120

**Published:** 2020-01-10

**Authors:** Chrystalla Antoniou, Rafaella Xenofontos, Giannis Chatzimichail, Anastasis Christou, Khosrow Kashfi, Vasileios Fotopoulos

**Affiliations:** 1 Department of Agricultural Sciences, Biotechnology and Food Science, Cyprus University of Technology, 3603 Lemesos, Cyprus; chrystalla.antoniou@cut.ac.cy (C.A.); rafaellaxen@gmail.com (R.X.); y.hadjimichael@theo.ac.cy (G.C.); 2 Agricultural Research Institute, Ministry of Agriculture, Rural Development and Natural Recourses, P.O. Box 22016, 1516 Nicosia, Cyprus; anastasis.christou@arinet.ari.gov.cy; 3 Department of Molecular, Cellular and Biomedical Sciences, Sophie Davis School of Biomedical Education, City University of New York School of Medicine, New York, NY 10031, USA; kashfi@med.cuny.edu; 4 Graduate Program in Biology, City University of New York Graduate Center, New York, NY 10016, USA

**Keywords:** alfalfa, hydrogen sulfide, aspirin, antioxidants, proline, nitric oxide, drought, priming

## Abstract

Land plants are continuously exposed to multiple abiotic stress factors like drought, heat, and salinity. Nitric oxide (NO) and hydrogen sulfide (H_2_S) are two well-examined signaling molecules that act as priming agents, regulating the response of plants to stressful conditions. Several chemical donors exist that provide plants with NO and H_2_S separately. NOSH is a remarkable novel donor as it can donate NO and H_2_S simultaneously to plants, while NOSH-aspirin additionally provides the pharmaceutical molecule acetylsalicylic acid. The current study aimed to investigate the potential synergistic effect of these molecules in drought-stressed *Medicago sativa* L. plants by following a pharmacological approach. Plants were initially pre-treated with both donors (NOSH and NOSH-aspirin) via foliar spraying, and were then subsequently exposed to a moderate water deficit while NO and H_2_S inhibitors (cPTIO and HA, respectively) were also employed. Phenotypic and physiological data showed that pre-treatment with NOSH synthetic compounds induced acclimation to subsequent drought stress and improved the recovery following rewatering. This was accompanied by modified reactive-oxygen and nitrogen-species signaling and metabolism, as well as attenuation of cellular damage, as evidenced by altered lipid peroxidation and proline accumulation levels. Furthermore, real-time RT-qPCR analysis revealed the differential regulation of multiple defense-related transcripts, including antioxidant enzymes. Overall, the present study proposed a novel role for NOSH compounds as efficient plant priming agents against environmental constraints through the coordinated regulation of multiple defense components, thus opening new horizons in the field of chemical priming research toward the use of target-selected compounds for stress tolerance enhancement.

## 1. Introduction

Abiotic stress factors represent major elements limiting global agricultural productivity [1]. The increased frequency of extreme environmental events resulting from undisputed climatic change significantly influences plant growth and development. Close examination of plant-to-plant communication in field conditions has revealed the development of unique strategies from plants as a response to abiotic stress factors, with one of the most intriguing being through priming for enhanced defence responses. The process of priming involves prior exposure to an abiotic or biotic stressor, thus rendering a plant more tolerant to future exposure [2]. An analogy therefore exists with the concept of vaccination in animals, where the administration of antigenic material results in the stimulation of adaptive immunity to a disease, and ultimately, the prevention or amelioration of the effects of pathogen infection. Although the process of priming has been known for decades, it has only recently been suggested that it can improve crop tolerance to environmental stressors in the field [3]. Priming can also be accomplished by applying natural or synthetic chemical compounds that act as signal transducers, thus “switching on” plant defence systems.

The use of chemical compounds as priming agents has indeed been shown to significantly enhance plant tolerance in various crop and non-crop species against a range of different individually applied abiotic stresses, with this field of study attracting increasing attention. Several types of molecules have the potential to act as a priming agent under specific conditions against a range of different environmental stressors [2]. A survey of relevant literature reveals a broad range, including amino acids (e.g., proline [4]), hormones (e.g., salicylic acid [5]), reactive oxygen-nitrogen-sulfur species (RONSS [6,7]), melatonin [8], and even water (i.e., hydropriming [9]). Reactive oxygen (e.g., H_2_O_2_), nitrogen (e.g., NO), and sulfur (e.g., H_2_S) species are of particular interest as they represent the key molecules involved in cellular signaling processes and gene regulation during stress and play a crucial role in the stress acclimation of plants [10,11]. Several reports have now led to the identification of a complex interaction between reactive oxygen species (ROS) and reactive nitrogen species (RNS), in which both reactive species are utilized by plants as signal transduction molecules during fundamental cellular and biological processes [10]; interestingly, recent reports have suggested that reactive sulfur species (RSS) may actually be additional players in this interaction [12].

NOSH-aspirin (NOSH-A; NBS-1120) is a novel hybrid synthetic compound belonging to the NSAID group that was first established as a high-potency compound for its anti-inflammatory action, e.g., anti-cancer [13,14,15] and neuroprotective activity in mammals [16]. NOSH-A is a donor that simultaneously releases nitric oxide (NO), hydrogen sulfide (H_2_S), and aspirin (acetylsalicylic acid), while NOSH donates NO and H_2_S. NOSH compounds represent very promising priming agent candidates due to this simultaneous donation, while they also avoid odor problems commonly associated with standard H_2_S donors, such as NaHS (smell of rotten eggs), thanks to its patented formulation. NO and H_2_S are two gasotransmitters with important physiological roles in plants, including their involvement in stress tolerance mechanisms [17]. Salicylates (e.g., salicylic acid) are produced by plants as part of their defense systems against biotic and abiotic stress conditions. Plant treatments with any of the three compounds strongly improved the germination and later plant growth in drought and salt stress conditions [17,18]. The aim of the present study was to examine the potential priming effects of NOSH and NOSH-aspirin pre-treatment in *Medicago sativa* plants growing under prolonged exposure to drought. A combinatorial physiological, biochemical, and molecular approach was employed toward this objective, while inhibitors of NO and H_2_S biosynthesis were also used, in order to decipher whether they present a cumulative protective role or act antagonistically.

## 2. Materials and Methods

### 2.1. Plant Material and Experimental Treatments

Alfalfa (*Medicago sativa* L.) seeds were generously obtained by the Cyprus National Genebank and Herbarium and sown after scarification in 7-cm diameter plastic pots (10 seeds per pot) filled with a mixture of sterile potting soil:perlite (3:1). Seeds were stratified in the dark for 4 days at 4 °C and subsequently transferred in a growth chamber at 22/16 °C day/night temperatures, at 60–70% RH, with a photosynthetic photon flux density of 100 μmol m^2^ s^−1^ and a 16/8-h photoperiod. Germinated seedlings were then thinned to three per pot. Growing plants were watered twice per week for the next 42 days until experimental treatments were applied. Plants were fertilized with commercial nutrient solution (Plant-Prod 20-20-20 Fertilizer, Lambrou Agro, Lemesos, Cyprus) every two weeks. In order to examine the effects of exogenous NOSH and NOSH-A application in plant tolerance to drought, plants were treated once via leaf spraying with 100 μM NOSH or NOSH-A (synthesized as previously described [13], and were a gift from Avicenna Pharmaceuticals Inc., New York, NY, USA) diluted in 30% (*v*/*v*) methanol till drip-off. Control samples were also sprayed with the same solvent. Drought stress conditions were achieved by withholding watering for 6 days, resulting in pronounced phenotypic foliar damage (in the form of chlorosis and leaf wilting). Samples were then rewatered to observe the recovery potential after 1 day (day 7). The optimal concentration, solvent, and method of application of NOSH and NOSH-A were chosen following preliminary experimental results where the hybrid donors were applied in gradient concentrations (data not shown). Overall, alfalfa plants were subjected to eleven treatments presented in detail in Table 1. Both inhibitors (cPTIO: NO inhibitor, HA: H_2_S inhibitor; Sigma-Aldrich, St. Louis, MO, USA) were applied at 100 μM based on the existing literature, either solo (for phenotypic observations shown in Supplementary Figure S1) or in mixture with NOSH synthetic compounds (for all measurements presented herein). Leaf samples were harvested both at day 6 (drought stress) as well as at day 7 (1 day after re-watering) to examine the recovery, flash-frozen in liquid nitrogen, and stored at −80 °C for subsequent analyses. Experiments were carried out in triplicate using pooled material (each replicate consisted of tissue harvested from a minimum of three independent plants).

### 2.2. Physiological and Biochemical Measurements

Stomatal conductance was measured in fully expanded leaves using a ΔT-Porometer AP4 (Delta-T Devices, Cambridge, UK) following the manufacturer’s instructions. Leaf chlorophyll fluorescence representing the photosystem II (PSII) maximum photochemical efficiency (*Fv*/*Fm*) was measured with an OptiSci OS-30p Chlorophyll Fluorometer (Opti-Sciences, Hudson, NH, USA). Leaves were dark-adapted for 30 min before fluorescence measurements were carried out. Lipid peroxidation levels, as a widely used cellular damage indicator, were determined from the quantification of malondialdehyde (MDA) content resulting from the thiobarbituric acid reaction, as previously described [19].

### 2.3. Reactive Species and Proline Quantification

Leaf hydrogen peroxide (H_2_O_2_) content was determined spectrophotometrically using potassium iodide (KI) as previously described [20]. Nitrite-derived nitric oxide (NO) content was quantified using the Griess reagent as previously described [21]. H_2_S content was determined following the methodology described by Nashef et al. [22]. Free proline content was measured with the ninhydrin reaction as described by Bates et al. [23]. Proline content was determined from a proline standard curve.

### 2.4. Antioxidant Enzyme Activities

Soluble proteins were extracted by homogenizing leaf samples (100 mg) in an ice-cold extraction buffer (0.1 M phosphate buffer pH 7.5, 0.5 mM EDTA, 1 mM PMSF). Each homogenate was centrifuged at 16,000× *g* at 4 °C for 20 min, and the supernatant was used for enzymatic activity and protein content assays. Protein content was determined according to the method of Bradford [24] using bovine serum albumin as a standard. Total superoxide dismutase (SOD) activity was assessed by measuring its ability to inhibit the photochemical reduction of nitro blue tetrazolium chloride (NBT), as proposed by Giannopolitis and Ries [25]. Catalase (CAT) activity was assayed by monitoring the H_2_O_2_ reduction by following the methodology of Aebi [26]. A detailed description of the methodology followed for the assay of both SOD and CAT enzymatic activity can be found in Filippou et al. [27]. All enzymatic activity assay results were expressed as specific activity units per milligram of protein.

### 2.5. RT-qPCR Analysis

Total RNA was extracted from leaves using TRIzol (TRI reagent; Sigma-Aldrich, St. Louis, MO, USA), followed by DNase digestion (RNase-free DNase Set; Qiagen, Hilden, Germany). The quality and quantity of RNA was analyzed spectrophotometrically using a Nanodrop 1000 Spectrophotometer (Thermo Scientific, Wilmington, DE, USA), while RNA integrity was checked using gel electrophoresis. For real-time RT-qPCR analyses, 1 μg of total RNA was converted into cDNA using a Primescript 1st Strand Synthesis Kit according to the manufacturer’s protocol (Takara, Shiga, Japan). Subsequently, real-time PCR was performed with a Biorad IQ5 (Biorad, Hercules, CA, USA). The reaction mix contained 4 μL cDNA in an RT buffer (diluted 1:5), 0.75 μM of each primer (see Table S1), and 1× master mix (SYBRGreen Super Mix, Invitrogen, San Diego, CA, USA). Reactions were carried out using three independent replicates. The thermocycler conditions were: initial denaturation at 95 °C for 5 min, followed by 40 cycles of amplification (95 °C for 30 s, annealing temperature (Ta; see Table S1) for 30 s, and 72 °C for 30 s), and a final elongation stage at 72 °C for 5 min. The relative quantification of transcript levels and the statistical analysis of RT-qPCR data (pairwise fixed reallocation randomization test) were performed using REST software in accordance with Pfaffl et al. [28]. *Actin11* was used as a housekeeping reference gene [29].

### 2.6. Statistical Analysis

Statistical analyses were carried out using SPSS v.11 (SPSS Inc., Chicago, IL, USA). Physiological and biochemical measurements were subjected to ANOVA, and then significant differences were determined between individual means using Duncan’s post-hoc pairwise comparison test at the 5% confidence level.

## 3. Results

### 3.1. Phenotypic Responses of Alfalfa Plants Pre-Treated with NOSH Synthetic Compounds Prior to Drought Stress Imposition

**Figure 1:**
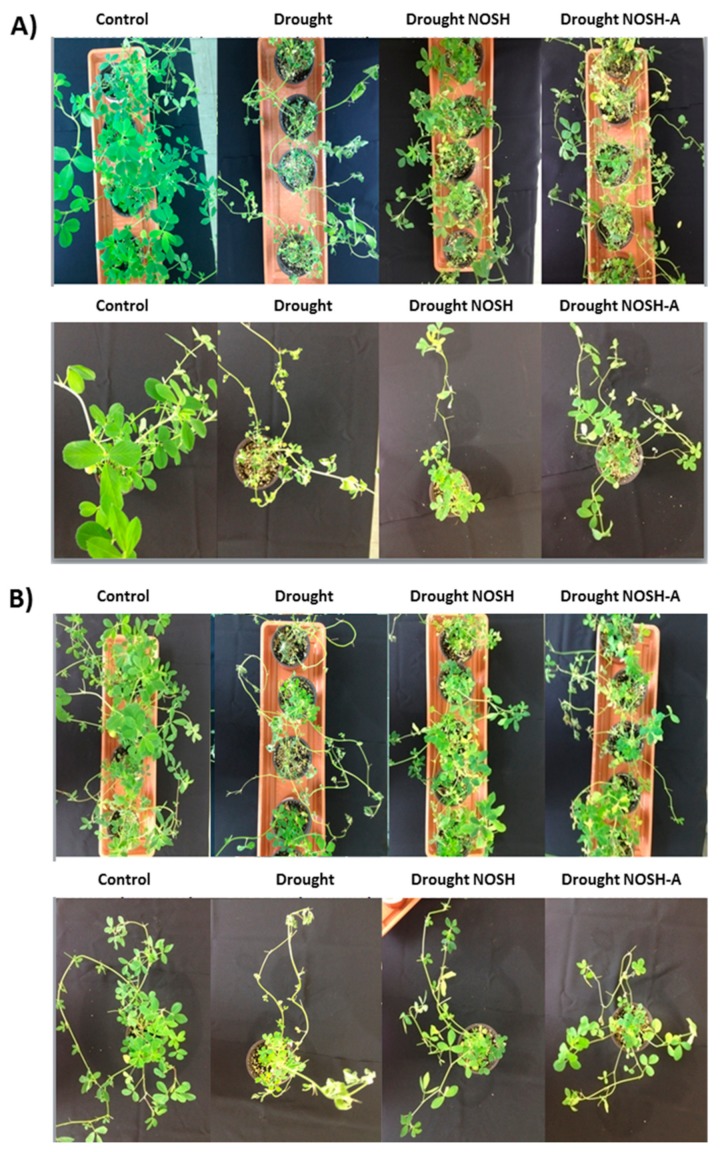
Phenotypic effects of NOSH and NOSH-aspirin (100 μΜ) pre-treatment on alfalfa plants (**A**) exposed to drought stress for 6 days by withholding watering, as well as (**B**) following rewatering for 1 day (day 7), with respective controls (MeOH-treated plants). Representative plants from selected treatments are shown. A full set of treatment phenotypes including inhibitors is shown in Supplementary Figure S1.

The macroscopic (phenotypic) observation of plants showed that NOSH and NOSH-A pre-treated plants that were subsequently drought-stressed had significantly improved vitality, turgor, and greening in comparison with drought-stressed plants. The latter demonstrated extensive chlorosis and loss of turgor that was indicative of stress-related damage at 6 days after stress imposition (Figure 1A).

Rewatering for 1 day revealed that NOSH and NOSH-A pre-treated samples had a notably improved recovery capacity compared with drought-stressed-alone samples; pre-treated plants retained turgor and greening, while stressed-alone plants showed extensive chlorosis and initial signs of necrosis (Figure 1B). Interestingly, plants were left to grow following rewatering for another 6 days without any further watering to replicate a second “wave” of drought stress. Phenotypic observations revealed that NOSH and NOSH-A pre-treated plants maintained their growth and vitality, while drought-stressed-alone plants showed extensive levels of tissue necrosis (data not shown).

### 3.2. Verification of the Successful Donation of Nitric Oxide and Hydrogen Sulfide Following NOSH and NOSH-A Pre-Treatment of Alfalfa Plants

**Figure 2:**
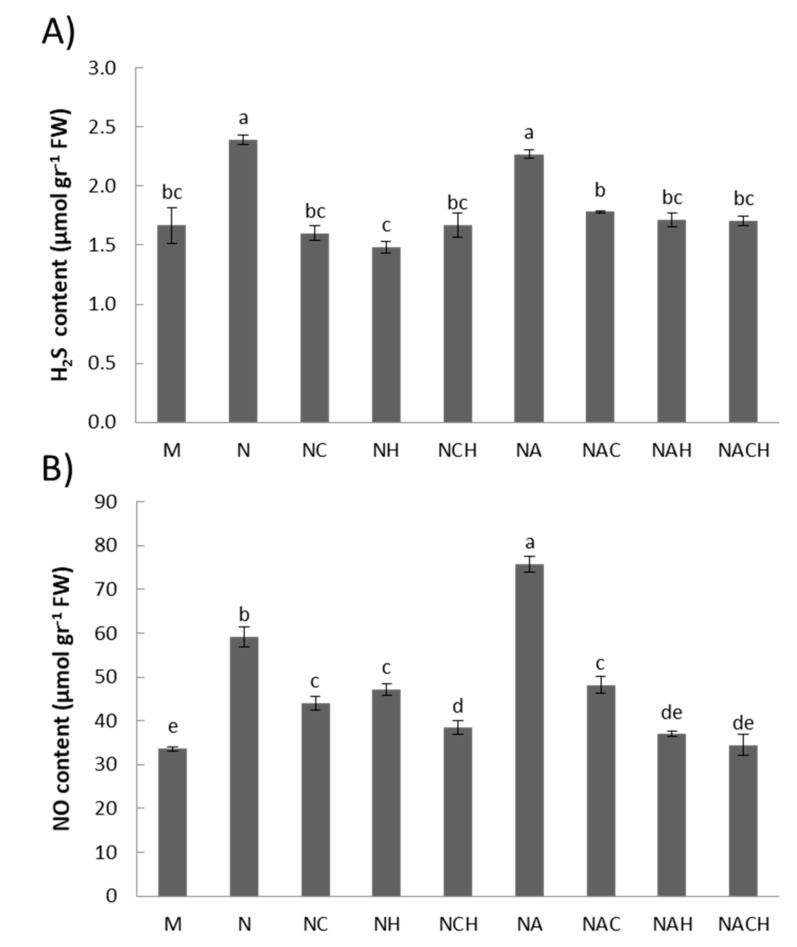
Quantification of (**A**) nitrite-derived NO content and (**B**) H_2_S content 1 day after application with NOSH synthetic compounds (100 μM) in alfalfa plants. Data are the means ± SE of three replications. Bars with different letters are significantly different (*p* < 0.05). Treatment abbreviations are explained in Table 1. FW: Fresh Weight.

The donations of nitric oxide and hydrogen sulfide following NOSH and NOSH-A pre-treatment in alfalfa plants was evaluated by quantifying the nitrite-derived NO content and H_2_S content after one day of hybrid donor application. Plants treated with either priming agent displayed significantly higher NO and H_2_S levels compared with the respective untreated samples, while use of appropriate inhibitors (cPTIO for NO, HA for H_2_S) resulted in a significant lowering of the reactive species content (Figure 2) indicative of successful compound donation.

### 3.3. Physiological and Cellular Damage Responses of NOSH and NOSH-A Pre-Treated Plants under Drought Stress

**Figure 3:**
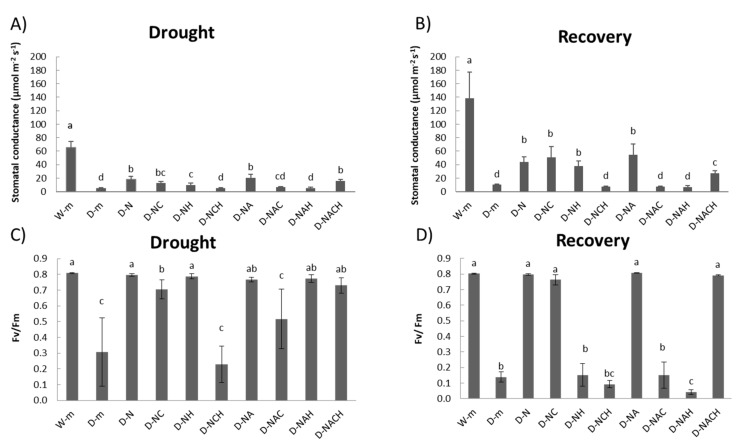
Effect of NOSH and NOSH-aspirin (100 μΜ) pre-treatment on the stomatal conductance (**A**,**B**) and chlorophyll fluorescence (**C**,**D**) of leaves of alfalfa plants exposed for 6 days either to drought stress by withholding watering (**A**,**C**), as well as following rewatering for 1 day ((**B**,**D**); day 7). Data are means ± SE of three replications. Bars with different letters are significantly different (*p* < 0.05). Treatment abbreviations are explained in Table 1.

Measurements of the physiological parameters indicated that drought-stressed plants had significantly lower stomatal conductance and chlorophyll fluorescence levels compared with watered plants, which was indicative of a stressed state. Contrarily, NOSH and NOSH-A pre-treated and subsequently stressed plants had significantly ameliorated stomatal conductance and chlorophyll fluorescence levels compared with drought-stressed samples (Figure 3A,C). Similar trends were also observed in the ability of plants to recover following rewatering (Figure 3B,D). The application of both inhibitors in NOSH-treated plants under drought stress conditions resulted in physiological parameters similar to ones recorded in unprimed, drought-stressed plants. Interestingly, the joint application of inhibitors in NOSH-A-treated plants did not reverse the protective effect, suggesting a key role of acetylsalicylic acid in the amelioration of drought-induced physiological damage.

**Figure 4:**
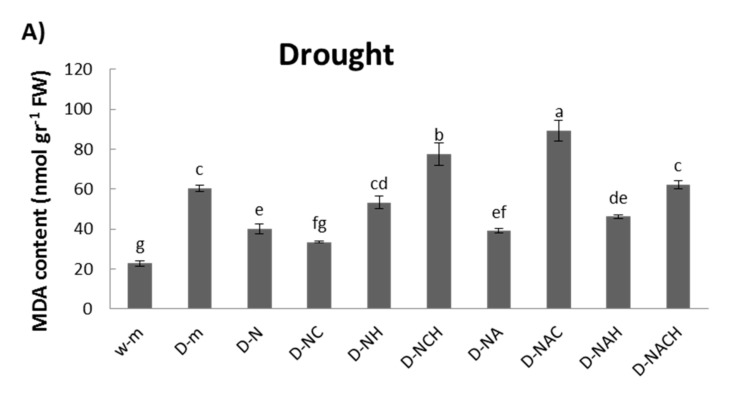

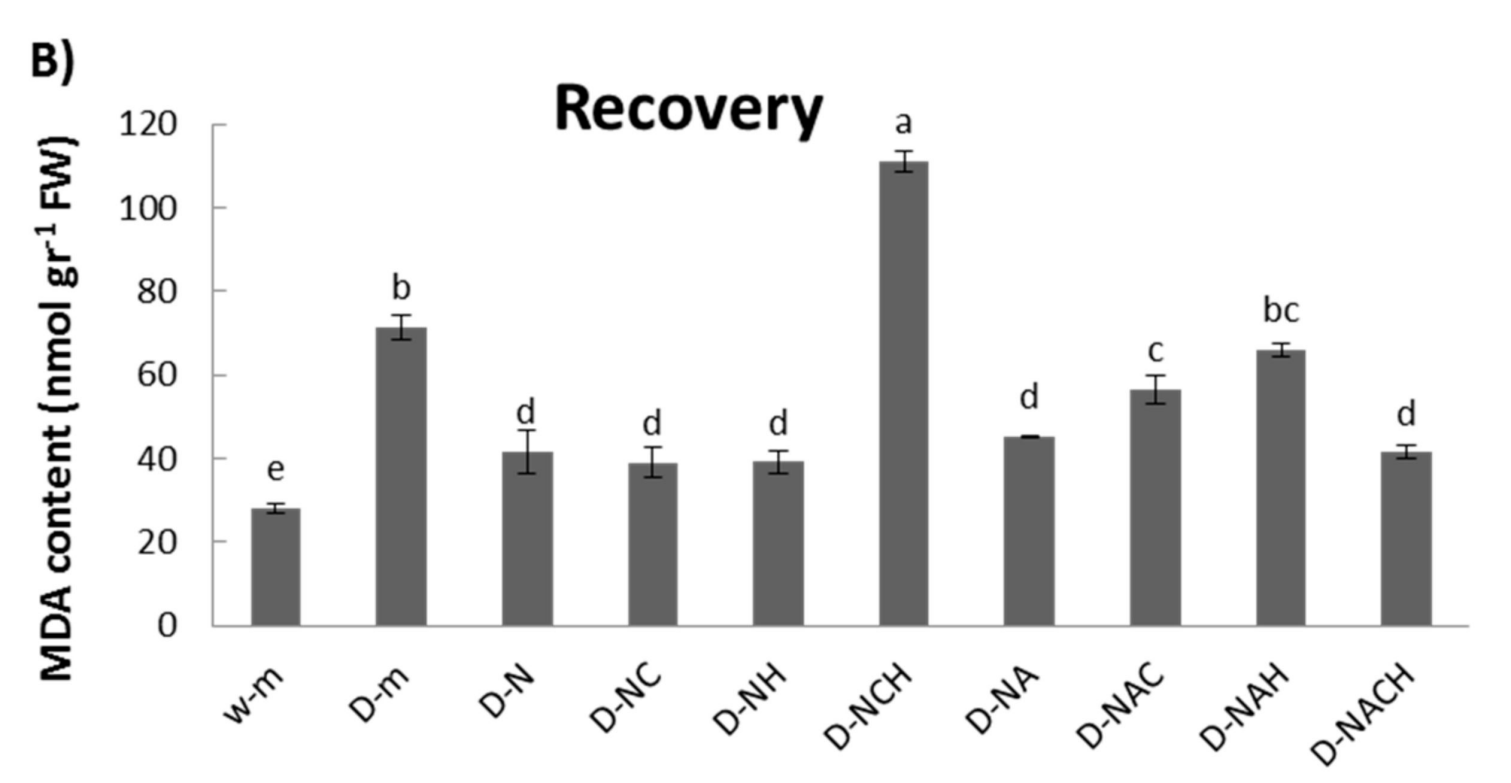
Effect of NOSH and NOSH-aspirin (100 μΜ) pre-treatment on the level of lipid peroxidation, measured as malondialdehyde (MDA) content, in the leaves of alfalfa plants (**A**) exposed for 6 days to drought stress by withholding watering, as well as (**B**) following rewatering for 1 day (day 7). Data are means ± SE of three replications. Bars with different letters are significantly different (*p* < 0.05). The treatment abbreviations are explained in Table 1. FW: Fresh Weight.

An examination of cellular damage levels was performed by means of the spectrophotometric determination of lipid peroxidation. Significant membrane damage was observed under drought conditions; however, the MDA content was lowered following NOSH and NOSH-aspirin pre-treatment, with both NOSH and NOSH-aspirin providing statistically significant protection in comparison with drought-stressed samples (Figure 4A). Interestingly, most treatments linked with NOSH and NOSH-A priming managed to recover significantly following rewatering in terms of lipid peroxidation levels, with the exception of plants treated with NOSH and both inhibitors, as well as plants treated with NOSH-A and HA (Figure 4B).

### 3.4. Regulation of Nitro-Oxidative Homeostasis in Alfalfa Plants under Drought Stress Following NOSH and NOSH-A Pre-Treatment

**Figure 5:**
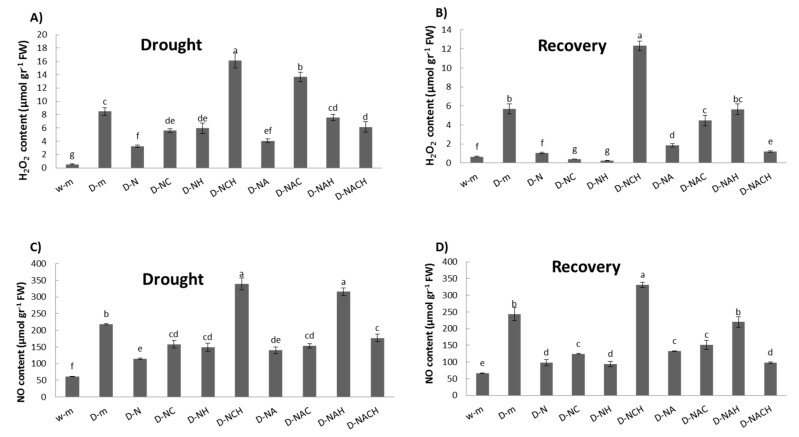
Effect of NOSH and NOSH-aspirin (100 μΜ) pre-treatment on (**A**,**B**) hydrogen peroxide, and (**C**,**D**) nitrite-derived NO content in leaves of alfalfa plants (**A**,**C**) exposed for 6 days to drought stress by withholding watering, as well as (**B**,**D**) following rewatering for 1 day (day 7). Data are means ± SE of three replications. Bars with different letters are significantly different (*p* < 0.05). Treatment abbreviations are explained in Table 1. FW: Fresh Weight.

Abiotic stress conditions are associated with increased levels of reactive oxygen (ROS) and reactive nitrogen (RNS) species content, which are toxic for the cells. During such conditions, the production of ROS and RNS exceeds the capacity of the antioxidative defense systems to remove them, causing nitro-oxidative stress. The quantification of H_2_O_2_ (major ROS) and nitric oxide (NO; major RNS) content revealed a massive induction in drought-stressed plants, which was significantly reversed in NOSH and NOSH-aspirin pre-treated plants compared with drought-stressed samples (Figure 5A,C). Similar patterns were recorded following rewatering of the plants (recovery phase; Figure 5B,D).

**Figure 6:**
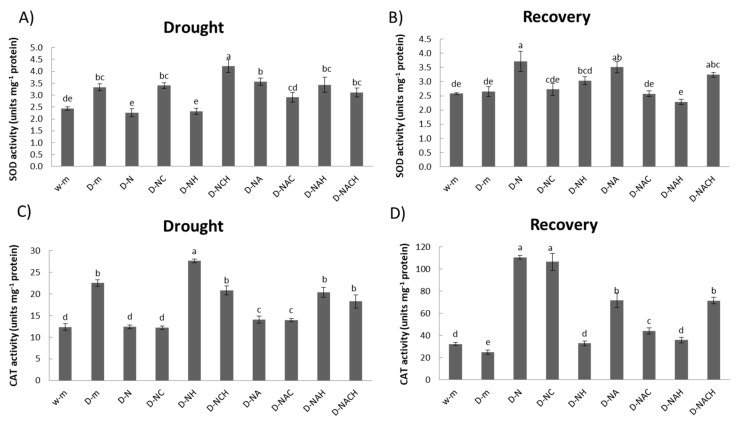
Effect of NOSH and NOSH-aspirin (100 μΜ) pre-treatment on superoxide dismutase (SOD; (**A**)) and catalase (CAT; (**C**)) enzymatic activity in leaves of drought-stressed *Medicago sativa* plants exposed for 6 days to drought stress by withholding watering, as well as ((**B**) SOD; (**D**) CAT activity) following rewatering for 1 day (day 7). Data are means ± SE of three replications. Bars with different letters are significantly different (*p* < 0.05). Treatment abbreviations are explained in Table 1.

The enzymatic activity of two key antioxidants (SOD and CAT) were evaluated. SOD dismutates superoxide radicals to hydrogen peroxide, while CAT directly scavenges hydrogen peroxide. Both enzymes appeared to be regulated in response to stress and priming agent pre-treatment in a differential manner (Figure 6A,C). Few specific enzymatic activity signatures were identified, with the main points being the significant induction in SOD and CAT activity in plants under drought stress compared with control ones, and the significant suppression of SOD and CAT activity following NOSH pre-treatment compared with unprimed, drought-stressed samples. The antioxidant activity profile under recovery (following rewatering) followed a reverse pattern, whereby NOSH and NOSH-A pre-treated plants had higher SOD and CAT activity levels compared with unprimed, drought-stressed plants (Figure 6B,D).

### 3.5. Proline Metabolism in Drought-Stressed Alfalfa Plants Following Priming with NOSH Synthetic Compounds

**Figure 7:**
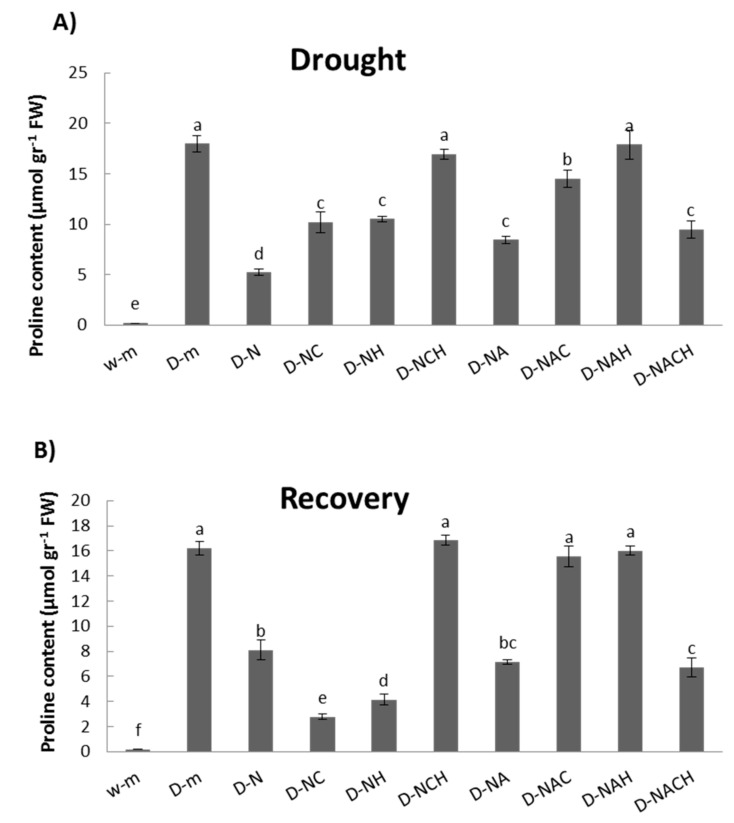
Effect of NOSH and NOSH-aspirin (100 μΜ) pre-treatment on proline content in the leaves of alfalfa plants (**A**) exposed for 6 days to drought stress by withholding watering, as well as (**B**) following rewatering for 1 day (day 7). Data are means ± SE of three replications. Bars with different letters are significantly different (*p* < 0.05). Treatment abbreviations are explained in Table 1.

Drought stress resulted in the significant accumulation of proline in leaf tissues compared with non-stressed control plants. The increase in proline content in plants pre-treated with NOSH and NOSH-A prior to drought stress imposition was significantly less pronounced compared with that of unprimed stressed plants (Figure 7A). Similar patterns were observed in general in plants under recovery following rewatering for 1 day, with the highest proline levels being recorded in unprimed, drought-stressed plants and NOSH pre-treated plants that were sprayed with both inhibitors and subsequently stressed (Figure 7B).

### 3.6. Molecular Characterization of Drought Stress Responses in NOSH and NOSH-A Primed Plants

**Figure 8:**
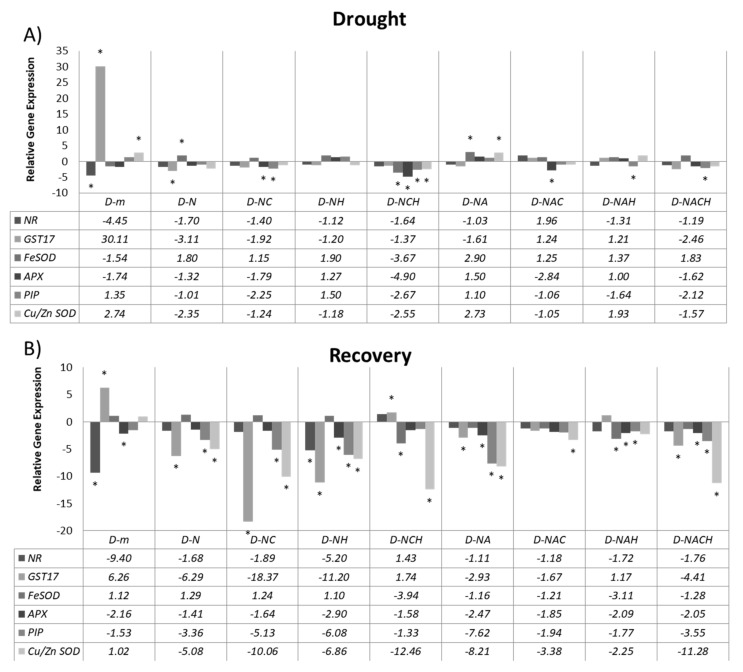
Effect of NOSH and NOSH-aspirin (100 μΜ) pre-treatment on the relative mRNA abundance (fold change relative to unprimed, unstressed samples) of selected transcripts encoding enzymes involved in reactive oxygen species (ROS) metabolism (*cAPX*, *Cu/ZnSOD*, *FeSOD*), detoxification (*GST17*), nitric oxide biosynthesis (*NR*), and water transport (*PIP*) in leaves of alfalfa plants (**A**) exposed for 6 days to drought stress by withholding watering, as well as (**B**) following rewatering for 1 day (day 7). Gene expression was assayed using RT-qPCR with three biological repeats. * *p* < 0.05, statistically different from control according to a pairwise fixed reallocation randomization test. Treatment abbreviations are explained in Table 1.

Finally, RT-qPCR gene expression analysis was carried out (Figure 8) for key defense-related genes, including major enzymatic antioxidants (*GST17*, *Cu/ZnSOD*, *FeSOD*, *cAPX*) linked with ROS scavenging, as well as genes involved in NO biosynthesis (*NR*). In addition, an aquaporin (*PIP*) was also examined, which is linked with the response to water deficit and transport. Interestingly, NOSH and primarily NOSH-A pre-treated plants under drought stress conditions demonstrated significant transcriptional upregulation for *SOD* isoforms, which are linked with the dismutation of superoxide radicals to H_2_O_2_ (Figure 8A). In addition, it should be noted that rewatering resulted in the general suppression of most defense-related genes examined in NOSH/NOSH-A pre-treated and subsequently stressed plants compared with both drought-stressed and rewatered plants, as well as primed and stressed plants prior to rewatering (Figure 8B), suggesting that the plants were in a better position to recover without the further transcriptional activation of defense-related genes, which is energy consuming for the plant.

## 4. Discussion

Water availability is thought to represent the most important environmental factor shaping plant evolution on earth [30]. Consequently, water deficit is one of the major constraints on agricultural production across ecosystems, thus rendering approaches toward mitigating its detrimental effects of prime importance from both a theoretical and applied point of view [31]. It is well-known that drought stress influences multiple plant metabolic pathways and induces a multitude of cellular responses [32]. Nevertheless, plants have developed an extensive defense apparatus, including valuable components, such as the antioxidant system, the accumulation of osmoprotective compounds, post-translational modifications, etc. [33]. In the present report, novel evidence is provided that highlights the protective effect of hybrid donor NOSH-aspirin against drought stress conditions in alfalfa plants. To our knowledge, this is the first report to show the cross-field application of synthetic anti-cancer compounds as plant-priming agents. Notably, these compounds have the added potential benefit of posing a low risk of negative environmental impact as they are donating very low amounts of natural molecules that already exist in plants.

The role of NO and H_2_S as key biological players acting as endogenous signaling molecules in plant stress responses is well documented [34]. This is also the case for salicylic acid, which has been long known to be heavily involved in an array of plant responses against abiotic stress factors [35]. Several reports exist that provide supporting information toward the protective effect of each molecule against drought stress when applied individually, in line with current findings. Such a protective function is the result of a coordinated orchestration of improved physiological performance, as well as upregulated antioxidant apparatus and other defense-related pathways.

Stress conditions lead to stomatal closure and limitations in photosynthetic capacity [36]. Similar to NOSH- and NOSH-A-primed plants under drought stress, the pre-treatment of strawberry plants with NaHS under polyethylene glycol (PEG) stress [19] and with sodium nitroprusside (SNP; NO donor) under NaCl stress [37] ameliorated chlorophyll fluorescence and stomatal conductance compared with unprimed, stressed samples, potentially leading to an improved maximum photochemical efficiency of photosystem II increased CO_2_ uptake and improved overall photosynthetic performance. Similarly, salicylic acid (SA) pre-treatment in wheat seedlings under osmotic stress increased their chlorophyll content [38], while SA supplementation to drought-stressed barley plants resulted in an increased net CO_2_ assimilation rate due to increased stomatal conductance, and eventually in increased plant dry mass [39]. The increased photosynthetic capacity was attributed to chloroplast biogenesis and upregulated photosynthetic enzyme expression following NaHS (H_2_S donor) application in spinach seedlings [40], while comprehensive proteomic analysis identified the upregulation of a number of proteins belonging to the photosynthetic apparatus following NaHS and SNP pre-treatment in drought-stressed citrus plants, which resulted in the maintenance of photosynthetic functionality and improved recovery of photosystem II under stress conditions [41].

Drought stress is associated with pronounced increases in reactive oxygen and nitrogen species (RONS) levels, which may lead to the significant oxidation of cellular components, so control of RONS and therefore of their metabolism is imperative under stress conditions [42,43]. Drought-induced cellular damage and the resulting loss of membrane integrity was alleviated in NOSH and NOSH-A pre-treated plants, as revealed by the significantly lower MDA content in primed, stressed plants compared with unprimed, drought-stressed plants. Interestingly, amelioration of lipid peroxidation was not as pronounced in plants pre-treated with NOSH-A and both inhibitors, suggesting that acetylsalicylic acid did not provide equal levels of stress protection as a solo application. Furthermore, the observed mitigation of stress-related cellular damage can be attributed at large to the restriction of drought-mediated nitro-oxidative stress, as evidenced by the lower RONS concentration in plants primed with NOSH compounds compared with unprimed ones under drought conditions. Notably, the highest levels of RONS indicative of nitro-oxidative damage were recorded in drought-stressed plants pre-treated with NOSH and both inhibitors, whereas drought-stressed plants pre-treated with NOSH-A and both inhibitors had lower levels of reactive species, suggesting that acetylsalicylic acid induced a certain degree of detoxification. These results are in agreement with those reported by Zhang et al. [44], who also reported that NaHS-pretreated and subsequently stressed plants had lower H_2_O_2_ levels, while Lisjak et al. demonstrated lower NO accumulation in *Arabidopsis* plants treated with NaHS [45]. Similarly, Christou et al. showed that NaHS [19] and SNP [37] pre-treatment managed to sustain both H_2_O_2_ and NO content in much lower concentrations in strawberry plants under PEG and salt stress, respectively, in comparison with unprimed, stressed plants.

Increased H_2_O_2_ content in untreated, stressed plants compared with primed and subsequently stressed plants correlates with the higher enzymatic activity of SOD, which dismutates superoxide radicals to H_2_O_2_ [36], in accordance with previous reports [46]. Contrarily, only partial correlation was observed between the total SOD enzymatic activity with corresponding gene expression levels since only one of the two SOD isoenzymes examined herein (*Cu/ZnSOD*) was induced in unprimed, stressed plants compared with control ones; meanwhile, both isoenzymes (*Cu/ZnSOD*, *FeSOD*) were significantly induced in NOSH-A pre-treated plants under stress, whereas no significant difference in the total SOD activity was observed between primed and unprimed plants under stress. The observed induction in *FeSOD* transcript levels in NOSH and NOSH-A primed plants under drought stress is in agreement with a recent report that demonstrated that treatment with 100 μM of NO donor SNP results in a significant induction of *FeSOD* in mature *Medicago truncatula* plants [47]. Any observed discrepancies in differences between antioxidant enzyme activities and transcript levels are likely due to organellar specificity or to the involvement of other unknown genetic factors that may regulate the expression of these genes [48], while the lack of correlation between H_2_O_2_ content and CAT activity (which scavenges H_2_O_2_ to oxygen and water [36] in NOSH and NOSH-A primed plants could be due to the complexity of the ROS scavenging system, which involves several other components (e.g., APX), as well as non-enzymatic antioxidants (e.g., ascorbic acid, tocopherols, and glutathione) [36].

It should be noted that a stress-related GST (*GST17*), known to act as a detoxifying molecule of oxidation products following their conjugation with GSH [49], was shown to be significantly induced following drought stress imposition, whereas expression was reversed to control levels in NOSH-A-primed plants or even suppressed in NOSH-primed ones. Previous work demonstrated the negative role of *AtGSTU17* in drought tolerance as knock-out plants displayed enhanced tolerance against drought [50], while *GST* suppression was suggested to take place as a result of plant pre-adaptation to subsequent stress factors, resulting in no further energy consumption needs for the activation of crucial metabolic defense-related pathways [51].

An important biochemical modification induced in plants toward stress protection is the accumulation of osmolytes [52]. Accumulation of the amino acid proline during drought stress improves adaptation by acting as a radical scavenger and osmoprotective agent [53]. The observed increase in proline content in drought-stressed plants is in accordance with previous findings [8], while NOSH and NOSH-A pre-treatment resulted in increased proline content in drought-stressed plants compared with controls, though to a significantly less pronounced extent compared with unprimed, stressed plants. The present findings suggest that NOSH and NOSH-A priming regulated proline homeostasis, which is crucial for plant growth at a low water potential as it may be used as an energy sink to sustain respiration needs under a water deficit [54] and to maintain high photosynthetic activity [55], ultimately leading to lower proline content in primed, stressed plants compared with unprimed, stressed ones due to a proline reservoir depletion.

Several lines of research suggest that signaling mechanisms in plant systems frequently do not operate as independent pathways, but that extensive cross-talk occurs between signal transduction pathways in a well-orchestrated manner [56]. In support of this observation, NOSH- and NOSH-A-priming activity against drought stress through the simultaneous donation of multiple signaling agents provides superior performance compared with individual components, as revealed through a comprehensive pharmacological inhibitor approach. Very few reports exist that have attempted to examine relevant potential synergistic effects, likely due to the complexity of the multifactorial experimental setup required. Even so, contradictory findings have been demonstrated; for example, the combination of NaHS and SNP did not reduce lipid peroxidation further than NaHS and SNP alone in Cd-stressed bermudagrass [57], whereas the combination of SNP with H_2_O_2_ resulted in a concentration-dependent interaction between them regarding heat stress responses in *Arabidopsis thaliana* [58] and protection against salt stress in basil plants [59]. In a similar fashion, the addition of SA or SNP partially reduced the toxic effects of Ni in canola plants, whereas Ni-stressed plants supplemented with SA + NO exhibited improved growth and photosynthetic pigment content [60]. Interestingly, treatment with H_2_S inhibitor HA in NOSH-aspirin pre-treated, drought-stressed plants in the present study resulted in lower cellular damage levels as indicated by an increased MDA content compared with plants treated with NO inhibitor cPTIO, supporting previous observations that H_2_S acts downstream of NO in abiotic stress responses [34]. Overall, the important research question on whether a combined application of priming agents could act synergistically needs further research in order to be fully elucidated.

## 5. Conclusions

In summary, the current findings demonstrated the capacity of NOSH synthetic compounds to provide significant protection in *Medicago sativa* plants against drought stress conditions, which resulted in major cellular damage and nitro-oxidative stress in affected plants. This protection appears to be achieved through a coordinated modification of improved physiological performance, reactive oxygen/nitrogen species homeostasis, and transcriptional regulation of defense-related pathways. Therefore, the significant potential for the use of NOSH compounds as priming agents (which has resulted in the Utility Patent Pub. No.: WO/2015/123273) is highlighted in terms of the tolerance enhancement of crop plants when challenged by adverse environmental conditions, with noteworthy beneficial impacts for agricultural production worldwide. Further experiments are being planned in an attempt to translate this technology to crops of agricultural importance, such as cereals, whereby agronomic parameters including yield and biomass will be evaluated, along with comprehensive systems biology approaches that will hopefully contribute to further understanding of NOSH compounds’ modus operandi in the regulation of plant priming responses under abiotic stress conditions.

## Figures and Tables

**Table 1:** Treatments carried out in the current experimental setup.

Treatment Abbreviation	Treatment
W-m	Control (methanol)
D-m	Drought (methanol)
D-N	Drought + NOSH
D-NC	Drought + NOSH + cPTIO
D-NH	Drought + NOSH + HA
D-NCH	Drought + NOSH + cPTIO + HA
D-NA	Drought + NOSH-A
D-NAC	Drought + NOSH-A + cPTIO
D-NAH	Drought + NOSH-A + HA
D-NACH	Drought + NOSH-A + cPTIO + HA

## Supplementary Materials

The following are available online at https://www.mdpi.com/2218-273X/10/1/120/s1, Table S1. Oligonucleotide primers used to amplify the targeted genes. Figure S1. Comprehensive set of all treatment phenotypes including inhibitors in (A) 6-day water-stressed and (B) recovered (6-day drought + 1-day rewatering) plants.
