# Supplementary material for: Exploring the Potential of Nitric Oxide and Hydrogen Sulfide (NOSH)-Releasing Synthetic Compounds as Novel Priming Agents against Drought Stress in Medicago sativa Plants
Source: Biomolecules. 2020 Jan 10;10(1):120. doi: 10.3390/biom10010120 (PMC7023404; doi:10.3390/biom10010120)
Supplement: Supplementary file 1 [file biomolecules-10-00120-s001.pdf]

Supplementary Materials

Exploring the Potential of Nitric Oxide and Hydrogen Sulfide (NOSH)-Releasing Synthetic Compounds as Novel Priming Agents against Drought Stress in *Medicago sativa* Plants

Chrystalla Antoniou [1], Rafaella Xenofontos [1], Giannis Chatzimichail [1], Anastasis Christou [2], Khosrow Kashfi [3,4] and Vasileios Fotopoulos [1,*]

Table S1. Oligonucleotide primers used to amplify the targeted genes.

	Gene	Primer	Nucleotide Sequence	Ta (°C)	Refence
1	*ACTIN11*	ACTIN11_FW	5'-ATG CCA TCC TTC GTC TTG A-3'	58	[1]
		ACTIN11_RV	5'-GCT GGT CCT GGC TGT CTC-3'		
2	*NR*	NR_FW	5'-GTAGGCCCCACAAAGGAGAA-3'	56	[2]
		NR_RV	5'-GAAGCGGGTGCAGTCGTAGA-3'		
3	*PIP*	PIP_FW	5'-GTTAAGGGCTTCCAACCACA-3'	56	TA29265_3880*
		PIP_RV	5'-TGCATTTCTCTTGGCATCAG-3'		
4	*Cu/ZNSOD*	Cu/ZnSOD_FW	5'-GTTTGTGCTGGTTCATCCAA-3'	60	[3]
		Cu/ZnSOD_RV	5'-CAGCAGACCTTTTCCCAGAC-3'		
5	*GST17*	GST17_FW	5'-GTTGGTAGGCTTTTGGGTGA-3'	60	[3]
		GST17_RV	5'-CTGCAATTGGTTTCTGAGCA-3'		
6	*cAPX*	APX-cyt_FW	5'-GGT CGC TTG CCT GAT GC-3'	56	[3]
		cAP-cyt_XRV	5'-CCA CCC AAC AAC TCC GTA AA-3'		
7	*FeSOD*	SODB3_FW	5'-TCT TGC AAC TGA GGA GGA C-3	52	[3]
		SODB3_RV	5'-AGG ACG CCG ATT CTG ATA-3'		

References

1. Mhadhbi, H.; Fotopoulos, V.; Mylona, P.V.; Jebara, M.; Aouani, M.E.; Polidoros, A.N. Role of antioxidant gene-enzyme responses in *Medicago truncatula* genotypes with different degrees of sensitivity to high salinity. *Physiol. Plantar.* **2011**, *141*, 201–214.
2. Antoniou, C.; Filippou, P.; Mylona, P.; Fasoula, D.; Ioannides, I.; Polidoros, A.N.; Fotopoulos, V. Developmental stage and concentration-specific sodium nitroprusside application results in nitrate reductase regulation and the modification of nitrate metabolism in leaves of *Medicago truncatula* plants. *Plant Signal. Behav.* **2013**, *8*, e25479.
3. Antoniou, C.; Chatzimichail, G.; Xenofontos, R.; Pavlou, J.J.; Panagiotou, E.; Christou, A.; Fotopoulos, V. Melatonin systemically ameliorates drought stress-induced damage in *Medicago sativa* plants by modulating nitro-oxidative homeostasis and proline metabolism. *J. Pineal Res.* **2017**, *62*, e12401.

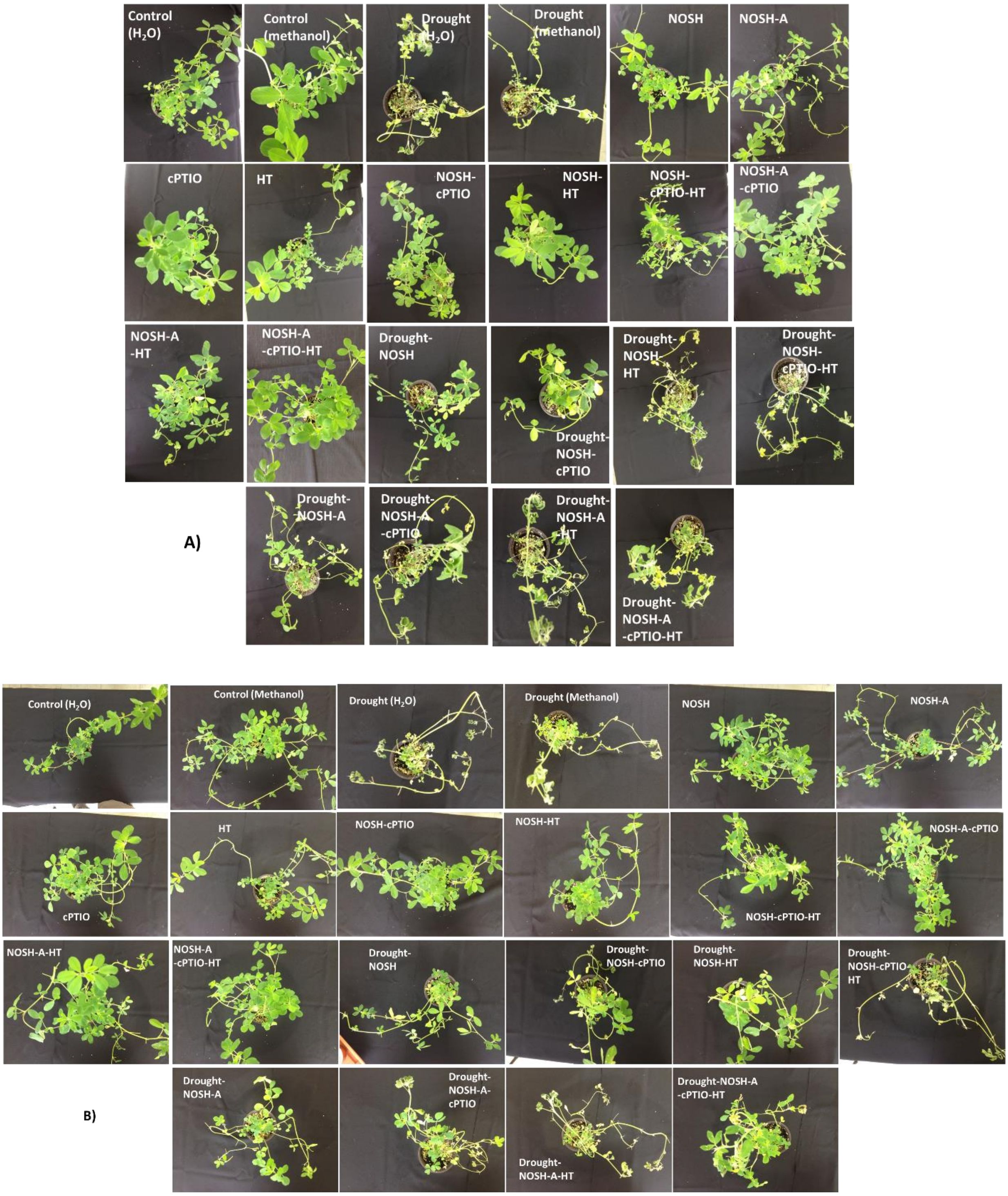

Figure S1. Comprehensive set of all treatment phenotypes including inhibitors in 6 d water-stressed (**A**) and recovered (6 d drought + 1 d rewatering; **B**) plants.

© 2020 by the authors. Licensee MDPI, Basel, Switzerland. This article is an open access article distributed under the terms and conditions of the Creative Commons Attribution (CC BY) license (http://creativecommons.org/licenses/by/4.0/).
